# Myotonometric and Postural Analysis in Patients with Post-Stroke Hemiparesis Included in a Rehabilitation Program: A Study Protocol

**DOI:** 10.3390/life15121791

**Published:** 2025-11-21

**Authors:** Constantin Ioan Covasala, Elena Constanta Amaricai, Sorana Sacui (Teaha), Anca Valentina Onciulenco, Alexandru Ianculescu, Cosmin Liviu Chifane, Nicoleta Flavia Roman, Catalin Nicolae Hreniuc

**Affiliations:** 1 Doctoral School, “Victor Babes” University of Medicine and Pharmacy, 300041 Timisoara, Romania; constantin.covasala@umft.ro (C.I.C.); soranateaha@yahoo.com (S.S.); nicoleta.roman.umfvbt@gmail.com (N.F.R.); 2 Faculty of Medicine, “Vasile Goldis” Western University, 310048 Arad, Romania; onciulencoa@gmail.com (A.V.O.); ianculescu_94_alexandru@yahoo.com (A.I.); chifanecosminliviu@gmail.com (C.L.C.); cata_hr@yahoo.com (C.N.H.); 3 Arad County Emergency Clinical Hospital, 310037 Arad, Romania; 4 Research Center for Assessment of Human Motion, Functionality and Disability, Department of Rehabilitation, Physical Medicine and Rheumatology, “Victor Babes” University of Medicine and Pharmacy, 300041 Timisoara, Romania

**Keywords:** myotonometry, gate analysis, stroke, spasticity

## Abstract

This observational study aims to assess both the posture and muscle tone characteristics of the upper and lower limbs in patients with hemiparesis following a cerebrovascular accident. Measurements will be taken comparatively between the affected and non-affected sides. The study will include patients with both ischemic and hemorrhagic strokes. Assessments will be conducted at months 1, 3, and 6 after the stroke event. In order to ensure the homogeneity of the study group, all patients will follow a physical exercise program tailored to their clinical stage of recovery. Myotonometric evaluation will be performed using the Myoton PRO Digital Palpation Device, which allows the assessment of muscle tone, elasticity, dynamic stiffness, tension state, relaxation time, and deformation ratio during muscle relaxation. Postural assessment will be conducted using the GaitON device by Auptimo. In addition to these instrumental evaluations, the following clinical scales will be applied: the Modified Ashworth Scale, Barthel Index, Instrumental Activities of Daily Living and the Modified Rankin Scale. Based on the three successive evaluations (at one, three, and six months after stroke event) we expect that with the spasticity decrease and the improvement of the posture of patients participating in the physical exercise program, both the objectively assessed parameters and the scores obtained from clinical scales will evolve in favor of better functioning.

## 1. Introduction

According to data from ALIA (a Romanian Association for Stroke Prevention), an estimated 60,000 to 70,000 strokes occur annually in Romania, with approximately 55,000 of these being ischemic strokes. Stroke is the second leading cause of death in Romania, after acute myocardial infarction [1]. Globally, stroke remains a major public health issue and one of the leading causes of long-term disability [2]. A primary contributor to post-stroke disability is the upper limb motor impairment, which significantly affects a person’s ability to perform daily activities [2]. When both upper and lower limb motor deficits are present, the impact on daily functioning is even more severe.

One of the most common reasons for reduced motor performance in patients with post-stroke hemiparesis is the alteration of muscle mechanical properties, particularly the tone, elasticity, and stiffness [2]. Although a variety of rehabilitation methods can be used to diminish the spasticity, objective measurement tools are essential for assessing their effectiveness [2].

The Modified Ashworth Scale is the most widely accepted and commonly used instrument for measuring increased muscle tone [3]. Spasticity, as defined by Jim Lance in 1980, is a velocity-dependent increase in muscle stretch reflexes, associated with increased muscle tone, as part of the upper motor neuron syndrome [3].

The study of Harb et al. on spasticity prevalence among stroke patients showed that 42.6% of patients developed spasticity, with 15.6% experiencing severe forms [3].

In addition to the Ashworth Scale, other tools such as the Barthel Index, IADL, and Modified Rankin Scale are used to broadly quantify stroke-related disability [4]. However, while imaging techniques like ultrasound and MRI can provide objective data on soft tissue properties [5], they are expensive, time-consuming, and impractical for routine or mobile clinical use.

In contrast, the myotonometer is a lightweight, portable device that allows rapid, objective, and reproducible muscle assessment in various clinical settings. It is handheld, easy to operate, and suitable for repeated measurements in different environments [6].

The study of Ng et al. quantified the elbow flexor and extensor stiffness in people with stroke. There was no significant difference in elbow muscle stiffness between the affected and non-affected arms. The patients suffered the stroke event at least 1 year ago [7]. We did not find any study we could use to assess and compare the myotonometer parameters of the affected side muscles in stroke patients in different rehabilitation stages.

Alongside muscle weakness and impaired motor control, spasticity represents a key component of the complex clinical presentation of upper motor neuron syndrome [8]. Collectively, these manifestations may lead to secondary complications such as pain and joint stiffness, further compromising the functional capacity of the affected limbs and ultimately limiting the performance of activities of daily living (ADLs) to varying degrees [8]. When spasticity occurs as an isolated manifestation and does not interfere with motor function, treatment may not be required. However, due to its potential to cause or contribute to significant functional impairment, accurate and early evaluation is essential.

A study by Jörg Wissel et al. (2010) [9] identified several risk factors associated with the development of post-stroke spasticity. These include the presence of paresis, greater paresis at week 16 after the event, a Modified Ashworth Scale (MAS) score ≥2 at week 6, involvement of more than two joints due to hypertonia, hemispasticity at week 6, and a lower baseline Barthel Index score [9]. Early identification of patients at high risk of severe spasticity is essential to ensure treatment initiation without delay [9].

With regard to posture, compared with healthy individuals, post-stroke patients commonly demonstrate a weight-bearing shift toward the unaffected side, leading to asymmetric postural alignment. This asymmetry alters gait, interferes with functional independence, and negatively affects patients’ daily activities and lifestyle [10]. Postural impairment in this population results primarily from paresis, which over time may lead to muscle atrophy and imbalances in spinal alignment [11]. Additionally, cognitive deficits, impaired proprioception, altered perceptual integration, and increased reliance on visual feedback further compromise postural stability and increase the risk of falls [12].

Regarding postural analysis in post-stroke patients, several clinical scales are employed. The Postural Assessment Scale for Stroke Patients (PASS) is particularly important [13]. This scale was specifically designed for assessing and monitoring posture in patients in this category [13]. Developed in 1999 as an adaptation of the Fugl–Meyer Balance Scale, the PASS consists of 12 items, scored from 0 to 3, with varying levels of difficulty to evaluate the ability to maintain or change posture in lying, sitting, or standing positions [13].

The GaitON device enables postural assessment in different settings and is user-friendly, making it suitable for evaluating all patients who are able to maintain an upright posture [14].

Study Objective: This observational study aims to evaluate muscle properties and posture-related parameters in patients with post-stroke hemiparesis (both ischemic and hemorrhagic), who are undergoing a structured physical exercise program. The main objective is to observe how changes in muscle properties and posture evolve in post-stroke hemiparetic patients and whether the data obtained from the myotonometer and the posture analysis system correlate with each other and with standard clinical scales. We expect to obtain data on how body posture changes depending on the dynamics of muscle parameters and how these factors influence each other in this category of patients. We assume that as spasticity improves, body posture will also improve, and that the data obtained using the devices will contribute to the selection of the most appropriate medical rehabilitation strategy.

Our study is based on the hypothesis that, in patients with acute post-stroke hemiparesis undergoing rehabilitation within the first six months, there is a correlation among the measurable muscle parameters assessed using the myotonometer and changes in body posture, the latter being objectively analyzed using the posture analysis system. Additionally, it is hypothesized that scores on clinical scales such as Barthel Index, Modified Rankin Scale, Modified Ashworth Scale, and IADL will evolve over time and show correlations with the data obtained through these objective measurement tools.

## 2. Materials and Methods

The study will include patients in the acute post-stroke phase, hospitalized in the Neurology Department of Arad County Emergency Hospital. The patients will be followed over a 6-month period, with assessments performed at one, three, and six months. All participants will undergo a rehabilitation program.

We chose to include both ischemic and hemorrhagic stroke patients in the study. In principle, the mechanism by which spasticity occurs is similar in both cases, as it is secondary to brain injury. However, we wanted to investigate whether there is a difference between the hemorrhagic and ischemic groups in terms of the results obtained through objective methods.

Measurements using the myotonometer will be performed to assess muscle tone, mechanical properties, and viscoelastic characteristics at the following sites:

- Upper limb: biceps brachii and triceps brachii.
- Lower limb: tibialis anterior and gastrocnemius muscles (medial and lateral heads).

Measurements will be taken bilaterally, comparing the affected and non-affected sides.

The clinical assessment tools will include the Barthel Index, Instrumental Activities of Daily Living (IADL), Modified Ashworth Scale, and the Modified Rankin Scale. At the end of the follow-up period, data from instrument-based measurements and clinical evaluations will be analyzed and correlated.

**Figure 1:**
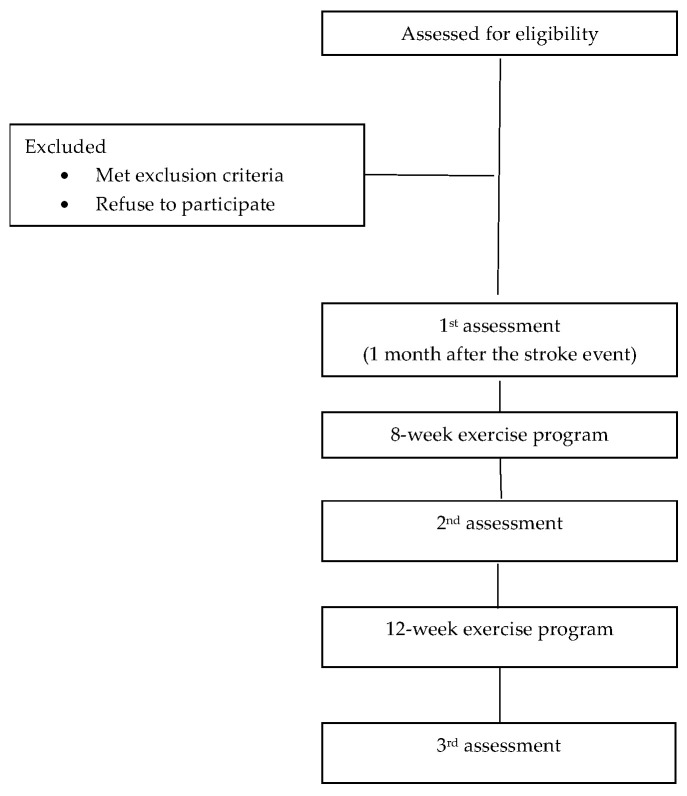
Flow chart of the study design.

The flowchart of the study design is presented in Figure 1.

### 2.1. Sample Size Calculation

The sample size was determined using data from the studies of Min-Jae and Dae-Sung, and calculations were performed using G*Power 3.1.9.7 (University of Kiel, Kiel, Germany). With an effect size of 0.5, a Type I error rate of α = 0.05, and a power of 0.8, the required minimum sample size was calculated to be 35 participants.

The within-group analyses will evaluate the effects over time. The evaluation of effect size was based on Cohen’s conventions for large effects [15]. The total sample size of our study (at least 35 patients) is in accordance with that calculated by Zielinski for patients who followed kinesiotherapy with the same statistical power and effect size (36 patients) [16].

### 2.2. Recruitment and Informed Consent

The study will include patients with post-stroke hemiparesis, both ischemic and hemorrhagic in origin. Prior to inclusion, comprehensive verbal and written information regarding the study protocol will be provided. Informed written consent will be obtained from all participants.

The study will be conducted in accordance with the principles outlined in the Declaration of Helsinki and has been approved by the Ethics Committee of the Victor Babeș University of Medicine and Pharmacy, Timișoara (Reference No. 74/16 December 2024).

#### 2.2.1. The Inclusion Criteria for Imaging-Confirmed Stroke Diagnosis (CT or MRI)

Eligible patients must meet the following criteria: aged 18 years or older; history of ischemic or hemorrhagic stroke; one-month post-stroke; able to maintain an upright posture for at least 10 min.

#### 2.2.2. Exclusion Criteria

The exclusion criteria for this study are morbid obesity; ongoing treatment with antispastic medication or botulinum toxin type A; and uncontrolled secondary hypertension.

Other exclusion criteria include recent surgical interventions preventing the patient from maintaining upright posture; lacunar infarcts in deep perforating arteries; limb or segmental amputations; patients with NYHA Class IV heart failure or dilated cardiomyopathy; patients with diagnosed dementia; severe balance disorders that prevent measurement in the required position; and severe aphasia preventing cooperation; post-traumatic orthopedic pathology of upper or lower limbs. Patients diagnosed with Parkinson’s disease are also ineligible to participate.

#### 2.2.3. Discontinuation Criteria

Patients who demonstrate noncompliance with the prescribed rehabilitation program will be excluded from the study. Additionally, participants who sustain injuries to the limbs during the study period that interfere with the accuracy of the measurements will also be withdrawn from the study.

### 2.3. Interventions: Exercise Program

The patients will follow a physical exercise program at home or in a rehabilitation center. Given that patients’ access to medical services varies, we considered that a standard rehabilitation program, whether performed at home or in a specialized center, would form a homogeneous study group. The physical exercise program was developed in collaboration with a certified physical therapist and is divided into four progressive stages.

Stage 1—Exercises for Mobility and Muscle Toning

Depending on each patient’s functional level, the program begins with passive mobilizations.

Goals:

- Improve muscle tone.
- Increase range of motion.
- Prevent postural deformities and joint contractures.

1. Supine Position:

- Passive ankle mobilization (flexion and extension): 2 sets of 10 repetitions per leg.
- Passive ankle rotation (circular movements): 2 sets of 10 reps in each direction.
- Passive knee mobilization (flexion and extension): 2 sets of 10 repetitions per leg.
- Passive foot mobilization (pushing the ankle toward the floor): 2 sets of 10 reps per leg.
- Passive arm mobilization (raising the arm above the head): 2 sets of 10 repetitions.

2. Side-Lying Position (on the non-affected side):

- Lifting the affected leg sideways: 2 sets of 10 repetitions.
- Knee flexion sideways: 2 sets of 10 repetitions per leg.
- Arm stretch (sideways): 2 sets of 10 repetitions.

3. Seated Position:

- Knee flexion and extension: 2 sets of 10 repetitions per leg.
- Toe standing for balance: 2 sets of 5 repetitions per leg.
- Arm mobilization (external rotation while seated or at bedside): 2 sets of 10 repetitions per arm.
- Finger stretching: 2 sets of 10 repetitions.

4. Standing Position:

- Assisted wall squats or frame-supported squats: 2 sets of 5 repetitions.
- Tiptoe standing (assisted): 2 sets of 5 reps per leg.
- Assisted single-leg stance: 2 sets of 3 repetitions per leg.

Session duration: 50 min.

Frequency: 5 sessions per week (daily, with rest on Saturdays and Sundays).

Stage 2—Active Mobilization and Muscle Strengthening

This stage focuses on active movement initiation and improving muscle strength and coordination. Patients begin controlling their movements independently, although some may still require therapist assistance.

Goals:

- Stretching of the posterior leg muscles.
- Strengthening of the anterolateral muscles.
- Enhancing joint mobility.
- Increasing muscle strength and endurance.

1. Supine Position:

- Active ankle flexion and extension: 2 sets of 10 repetitions.
- Active ankle rotation (clockwise and counter-clockwise): 2 sets of 10 reps in each direction.
- Active knee flexion and extension: 2 sets of 10 repetitions.
- Active ankle pushing toward the floor: 2 sets of 10 repetitions per leg.
- Active arm movement above the head: 2 sets of 10 repetitions.

2. Side-Lying Position:

- Active leg lift (sideways): 2 sets of 10 repetitions.
- Active knee flexion (sideways): 2 sets of 10 repetitions per leg.
- Active arm stretching (sideways): 2 sets of 10 repetitions.

3. Seated Position:

- Active knee flexion and extension: 2 sets of 10 repetitions per leg.
- Active toe lifts: 2 sets of 10 repetitions per leg.
- Active external arm rotation: 2 sets of 10 repetitions per arm.
- Active finger extension: 2 sets of 10 repetitions.

4. Standing Position:

- Active wall squats: 2 sets of 5 repetitions.
- Active tiptoe standing: 2 sets of 5 repetitions per leg.
- Active single-leg stance: 2 sets, maintaining balance for 2 s per leg.

Session duration: 50 min.

Frequency: 5 sessions per week (daily, with rest on Saturdays and Sundays).

Stage 3—Coordination and Functional Movement Training

This stage emphasizes coordination between upper and lower limbs and postural control during functional movements. Patients begin to perform tasks that simulate daily activities.

Goals:

- Improve coordination of the affected upper and lower limbs.
- Enhance balance during functional tasks.

1. Supine Position:

- Active leg lifts (hip and knee flexion): 2 sets of 10 repetitions per leg.
- Active ankle flexion and extension with resistance: 2 sets of 10 repetitions.
- Active leg abduction (sideways lifting): 2 sets of 10 repetitions per leg.
- Active overhead arm movement (shoulder flexion): 2 sets of 10 repetitions.

2. Side-Lying Position (for toning and coordination):

- Lateral leg raises (active, with pelvic stability control): 2 sets of 10 repetitions.
- Active arm abduction (side-lying): 2 sets of 10 repetitions per arm.
- Active knee flexion (side-lying): 2 sets of 10 repetitions.

3. Seated Position (for balance and coordination):

- Active knee flexion and extension (simulated walking in place): 2 sets of 10 repetitions per leg.
- Tiptoe raises with weight shifting: 2 sets of 10 repetitions per leg.
- Active external arm rotation (arm-leg coordination): 2 sets of 10 repetitions per arm.
- Active finger stretching while seated: 2 sets of 10 repetitions.

4. Standing Position (for functional balance and coordination):

- Deep squats (supported by wall or walker): 2 sets of 8 repetitions.
- Tiptoe raises with weight shifting: 2 sets of 5 repetitions per leg.
- Single-leg stance with weight transfer: 2 sets of 10 s per leg.

Session duration: 45 min.

Frequency: 5 sessions per week (daily, with rest on Saturdays and Sundays).

Stage 4—Functional Mobility and Dynamic Balance

This stage aims to improve coordination, balance, and functional mobility.

1. Supine Position:

- Active leg lifts (controlled hip and knee flexion): 2 sets of 10 repetitions per leg.
- Circular foot slides on the floor (to tone leg and hip muscles): 2 sets of 10 repetitions per leg.
- Active arm movement (shoulder flexion and extension): 2 sets of 10 repetitions.
- Side-to-side leg movements (abduction and adduction): 2 sets of 10 repetitions per leg.

2. Side-Lying Position (for lateral mobility and stability):

- Controlled lower limb abduction with brief hold: 2 sets of 10 repetitions per leg.
- Simultaneous active abduction of arm and lower limb: 2 sets of 10 repetitions per limb.
- Active knee flexion and extension: 2 sets of 10 repetitions.

3. Seated Position (for functional coordination and balance):

- Active knee flexion and extension: 2 sets of 10 repetitions per leg.
- Tiptoe raises with weight shifting (stability training): 2 sets of 10 repetitions per leg.
- Marching in place with arm movements (interlimb coordination): 2 sets of 2 min.

4. Standing Position (for dynamic balance and functional movement):

- Marching in place with single-leg support: 2 sets of 1–2 min.
- Deep squats with weight transfer (stability training): 2 sets of 8 repetitions.
- Tiptoe walking (to mobilize the ankle and improve balance): 2 sets of 2 min.

Session duration: 40 min.

Frequency: 5 sessions per week (daily, with rest on Saturdays and Sundays).

### 2.4. Measurements

Patients with post-stroke hemiparesis will be assessed at 1, 3, and 6 months post-ictal event using the following evaluation tools:

1. Myotonometric Evaluation

Muscle tone and biomechanical properties will be assessed in upper limbs (biceps brachii and triceps brachii) and lower limbs (tibialis anterior and gastrocnemius-medial and lateral heads).

Device used: Myoton PRO Digital Palpation Device—Software v.5.0.0.232 (Myoton, Estonia) [6]

Positioning for Upper Limb Assessment:

- Biceps brachii: Patient in supine position, arm relaxed alongside the trunk, forearm in neutral position. A towel is placed under the wrist for comfort. Measurement point: long head, lateral side of the muscle, mid-belly.
- Triceps brachii: Patient in side-lying position, arm relaxed. Measurement point: medial head of the triceps, mid-belly.

Positioning for Lower Limb Assessment:

- Tibialis anterior: Patient in supine position, relaxed. Measurement taken on the anterior compartment, at the upper third of the line connecting the tibial tuberosity and lateral malleolus.
- Gastrocnemius (medial and lateral): Patient in prone position, relaxed.
  - Lateral head: measured at the upper third of the line from the popliteal fold to the lateral malleolus.
  - Medial head: measured at the upper third of the line from the popliteal fold to the medial malleolus.

Evaluation type: Left/right comparison between the affected and unaffected sides.

Duration per procedure takes approximately 5 min.

The device operates by recording the damped natural oscillations of soft tissue in response to a brief, low-force mechanical impulse with controlled preload. The MyotonPRO provides quantitative analysis of tissue properties through five parameters: muscle tone, elasticity, dynamic stiffness, relaxation time, and creep (deformation ratio during relaxation phase).

2. Postural Analysis

GaitON v1.1.0—Auptimo Technologies LLP (Delhi, India) is the device that will be used for postural analysis [13].

Postural analysis is performed using adhesive markers applied to anatomical landmarks, followed by front, rear, and left/right profile photographs. These images are analyzed via specialized software that interprets the data.

The markers will be placed bilaterally on the acromion, anterior superior iliac spine (ASIS), center of the patella and also to the tibial tuberosity for the anterios view. For the posterior view, the markers are going to be placed bilaterally on the calcaneus, Achilles tendon insertion, mid-portion of the Achilles tendon, at the level of the medial malleolus, and 15 cm above that point on center of the calf. For lateral view (both side left and right) the adhesive markers will be placed on the following landmarks: the spinous process of C7 vertebra, the center of the humeral head, the greater trochanter, the lateral femoral condyle, and the lateral malleolus [14]. The evaluation lasts approximately 5 min.

Following the upload of photographic captures into the software, it generates a report containing information about physiological postural deviations, separately analyzed for each anatomical plane. For the anterior view, the software provides data on lateral head tilt, shoulder drop, lateral trunk sway, lateral pelvic drop, and Q-angle. In the posterior view, it evaluates rear foot eversion or inversion. The lateral view (left and right) highlights the forward head angle, shoulder protraction, and genu recurvatum [14,17].

3. Completion of clinical scales: Barthel Index, IADL, Modified Ashworth Scale, and Modified Rankin Scale (all validated in Romanian).

The Barthel Index measures the patient’s performance in ten activities of daily living based on the level of external assistance required. The maximum score is 100 points, representing full autonomy. A score of 60 points indicates “assisted independence,” while a score of 75 corresponds to “quasi-independence” (source: original form). The ten key domains essential for an independent life include feeding, bathing, personal hygiene, dressing, bowel control, bladder control, toilet use, transferring to and from bed or chair, walking, and stair climbing [18]. This scale was developed over a ten-year period, from 1950 to its publication in 1964, allowing nurses to evaluate the self-care abilities of patients with neuromuscular and musculoskeletal conditions [18]. The main advantage of the Barthel Index is its simplicity, making it useful for assessing a patient’s level of independence before treatment, tracking progress during rehabilitation, and establishing functional status upon reaching the maximum benefit [19].

The IADL Scale (Lawton Scale for Instrumental Activities of Daily Living) was first published in 1969 in *The Gerontologist* [20,21,22,23]. It is a questionnaire consisting of eight items assessing the ability of older adults to carry out more complex daily activities. The score is calculated by summing the values assigned to each question [21,22,23]. A maximum score of eight indicates that all activities can be performed independently. A minimum score of zero means that the activities assessed can only be performed partially or not at all [21,22,23].

The Modified Ashworth Scale is the commonly used clinical tool for assessing increased muscle tone [3].

The impact of severe spasticity on a patient’s life is substantial, affecting everything from daily activities to mental health and even income [3].

However, spasticity may also be beneficial for patients with weak limbs, especially in the lower extremities, allowing them to transfer or move with less assistance [3]. For these reasons, evaluating spasticity is important so that clinicians can determine whether their therapeutic interventions are effective [3].

In 1964, Bryan Ashworth published the original Ashworth Scale as a method for classifying spasticity in patients with multiple sclerosis. The original version was a 5-point numerical scale grading spasticity from 0 (no resistance) to 4 (limb rigid in flexion or extension) [3,24]. In 1987, while conducting a study to assess inter-rater reliability in manual testing of elbow flexor spasticity, Bohannon and Smith modified the scale by adding a “1+” level to increase sensitivity [3,25]. Since then, the Modified Ashworth Scale (MAS) has been widely used in both clinical practice and research as a standard measurement of spasticity [3,25].

The Modified Rankin Scale (mRS) is a widely used tool for measuring the degree of disability or dependence in daily activities among individuals who have experienced a stroke or other causes of neurological impairment. It has become the most commonly applied outcome measure in clinical trials related to stroke. Originally introduced in 1957 by Dr. John Rankin, the scale was later revised into its current accepted format by Prof. C. Warlow’s team study in the late 1980s [4,26].

The scale ranges from 0 to 6, with 0 indicating perfect health without symptoms and 6 indicating death: 0. No symptoms; 1. No significant disability. Able to carry out all usual activities despite some symptoms; 2. Slight disability. Able to look after own affairs without assistance but unable to perform all previous activities; 3. Moderate disability. Requires some help but able to walk unassisted; 4. Moderately severe disability. Unable to attend to own bodily needs without assistance and unable to walk unassisted; 5. Severe disability. Requires constant care and attention, bedridden, incontinent; 6. Dead.

### 2.5. Statistical Analysis

The descriptive statistics will be computed for all variables (mean and standard deviation). Before statistical applications, the normal distribution of values will be verified by the D’Agostino–Pearson normality test. The intragroup data (myotonometer and postural analysis parameters at baseline and after the physical exercise program, namely at 3 months and 6 months, respectively, after the stroke event) will be compared with the paired *t*-test. The myotonometer and postural analysis parameters of the affected and non-affected side will be compared with Student’ s unpaired *t*-test or a Chi-squared test. A *p*-value less than 0.05 will be considered statistically significant.

## 3. Expected Results

Following data collection through measurements and clinical scale evaluations, we expect to find a possible correlation between postural changes and the measurable parameters obtained with the myotonometer in patients with post-stroke hemiparesis. Furthermore, we anticipate that posture deficits and muscle properties will be reflected in the clinical scale scores. The results will contribute to a better understanding of the relationship between posture and spasticity and may influence the therapeutic strategies chosen by neurologists and rehabilitation physicians in the complex recovery process of these patients.

## 4. Discussion

To our current knowledge, this is the first study to investigate whether there is a correlation among myotonometric parameters and postural analysis in patients with post-stroke hemiparesis included in a rehabilitation program. Evaluations are scheduled for months 1, 3, and 6 months post-stroke. Furthermore, the data obtained from the instrumental measurements will be compared with the scores on the clinical scales (Barthel, IADL, Modified Ashworth, and Modified Rankin).

With this handheld device, the MyotonPRO Digital Palpation Device, five key muscle parameters are measured, namely tone, stiffness, elasticity, relaxation time, and creep [6]. Myotonometry allows for the assessment of muscle tension (at rest), biomechanical properties (dynamic stiffness and logarithmic decrement, which reflect elasticity and the damping of oscillation), and viscoelastic properties (stress relaxation time and the ratio of relaxation to deformation time, which characterize creep) [6,27].

As in other studies, we selected muscles from the upper limbs (biceps and triceps brachii) and from the lower limbs (superficial muscles: gastrocnemius and tibialis anterior), with evaluations of affected and non-affected parts, comparing the healthy and affected sides, as well as agonist and antagonist muscle groups [2,28,29]. The biceps brachii functions primarily in elbow flexion and forearm supination [30,31,32]. The medial head of the triceps brachii, on the other hand, is active during forearm extension regardless of supination or pronation [30]. In patients with post-stroke hemiparesis, daily living activities are impaired due to brachial motor deficits, secondary to muscle weakness and the presence of abnormal synergy patterns [33]. Moreover, restriction of movement in the affected arm is frequently observed due to musculoskeletal changes caused by prolonged immobilization in the chronic phase, which affects the overall functionality of the upper limb in everyday use [33].

The tibialis anterior is the most medial and the largest muscle in the anterior compartment of the lower limb [30,32]. Its primary actions are dorsiflexion and foot inversion, and it also functions as an important stabilizer of the ankle [30,32,34,35,36]. For these reasons, this muscle plays a vital role in everyday activities such as walking, kicking, and hiking [30,32]. The gastrocnemius muscle is a superficial muscle located just beneath the skin in the posterior compartment of the calf. Its main function is the plantar flexion [37,38]. It is crucial for propulsion and is actively involved in walking, running, and jumping [37,38,39].

The GaitON device has already demonstrated its reliability in posture analysis, as shown in a study published in October 2024, conducted by Alam et al. [14]. It has also proven to be a valuable tool for tracking biomechanical changes over time, as it is capable of detecting even the smallest postural changes [14]. Its applications are relevant in medical rehabilitation, sports science, and various clinical settings. The data obtained from postural analysis guide healthcare professionals in selecting appropriate rehabilitation techniques, with the ultimate goal of reducing recovery time [14].

Stroke remains one of the leading causes of disability, ranking third globally, affecting approximately 15 million people per year worldwide [40,41,42]. Of the 10 million stroke survivors, around 5 million live with significant long-term disability [26]. Moreover, statistics show that over 100 million people globally have experienced or are currently living with cerebrovascular disease [43], and 1 in 4 individuals over the age of 25 is expected to suffer a stroke during their lifetime [43]. As for spasticity, it is known to occur in 4–42.6% of post-stroke cases and can be present even in the early phase after ischemic stroke [1], typically involving the flexor muscles of the upper limbs and the extensor muscles of the lower limbs. Structural changes in muscles and tendons caused by spasticity impair motor control of the affected limb, leading to delayed or incomplete recovery and ultimately contributing to long-term disability.

To the best of our knowledge, this is the first study to investigate whether there is a correlation between muscle parameters measurable with the Myoton device and postural analysis in patients with stroke. Furthermore, it explores whether the results obtained through objective methods correlate with clinical scales. These facts represent a strength of our study.

## 5. Conclusions

Based on the three successive evaluations (at one, three, and six months after stroke event) we expect that with a spasticity decrease and improvement in the posture of patients participating in the physical exercise program, both the objectively assessed parameters and the scores obtained from clinical scales will evolve in favor of a better functioning. Research using these instruments helps us to better understand the physiological changes occurring after a stroke, providing guidance for rehabilitation and neurology specialists in choosing appropriate methods and techniques.

## Data Availability

No new data were created or analyzed in this study.
